# Antisense epidermal growth factor receptor RNA transfection in human glioblastoma cells down-regulates telomerase activity and telomere length

**DOI:** 10.1038/sj.bjc.6600244

**Published:** 2002-04-22

**Authors:** X-X Tian, JC-S Pang, J Zheng, J Chen, S S T To, H-K Ng

**Affiliations:** Department of Pathology, Health Science Center, Peking University, Beijing, China; Department of Anatomical and Cellular Pathology, Prince of Wales Hospital, The Chinese University of Hong Kong, Hong Kong, China; Department of Pathology, Peking Union Medical College Hospital, Beijing, China; Department of Nursing and Health Sciences, The Hong Kong Polytechnic University, Hong Kong, China

**Keywords:** glioblastoma, epidermal growth factor receptor, antisense, telomerase

## Abstract

Epidermal growth factor receptor is overexpressed and/or amplified in up to 50% of glioblastomas, suggesting an important role of this gene in glial tumorigenesis and progression. In the present study we demonstrated that epidermal growth factor receptor is involved in regulation of telomerase activity in glioblastoma. Antisense-epidermal growth factor receptor approach was used to inhibit epidermal growth factor receptor expression of glioblastoma U87MG cells. Telomerase activity in antisense-epidermal growth factor receptor cells decreased by up to 54 folds compared with control cells. Moreover, the telomere lengths of antisense-epidermal growth factor receptor cells were shortened. In addition, the tumorigenicity of antisense-epidermal growth factor receptor cells was significantly inhibited. Taken together, there were strong correlations between tumorigenicity and epidermal growth factor receptor expression levels, and between tumorigenicity and telomerase activity. These results provide evidence that epidermal growth factor receptor plays an important role in the regulation of telomerase activity of glioma cells. Our findings provide new insights into both the biological functions of epidermal growth factor receptor and the regulation of telomerase activity. The inhibition of telomerase activity triggered by antisense-epidermal growth factor receptor treatment may reflect yet another mechanism of antisense-epidermal growth factor receptor approach in tumour suppression.

*British Journal of Cancer* (2002) **86**, 1328–1332. DOI: 10.1038/sj/bjc/6600244 www.bjcancer.com

© 2002 Cancer Research UK

## 

The epidermal growth factor receptor (EGFR) is a transmembrane glycoprotein of 170 kDa and is composed of an extracellular ligand-binding domain, a single hydrophobic membrane-spanning domain and a cytoplasmic tyrosine kinase domain (Yamada *et al*, 1997). In the EGFR-Ras signalling pathway, the binding of ligands to EGFR activates Ras, which then activates Raf. The activated Raf kinase can initiate the mitogen activated protein (MAP) kinase cascade. The activated MAP kinase then migrates into the nucleus and there phosphorylates many transcription factors such as c-*myc*, c-*jun*, and c-*ets*, which affect gene expression and ultimately cell growth (Yamada *et al*, 1997; Tang *et al*, 1997).

In addition to serving a normal physiological function, aberrant expression of EGFR has also been linked with a number of pre-malignant or malignant diseases, including benign hyperplasia of the skin, mammary carcinoma, glioblastoma, and hepatic carcinoma (Khazaie *et al*, 1993). In glioblastoma, EGFR is found to be overexpressed and /or amplified in up to 50% of all cases, suggesting an important role of this gene in glial tumorigenesis and progression (Ng and Lam, 1998). The use of antisense-EGFR RNA to block the function of enhanced EGFR in human glioblastoma cells has been reported to inhibit cellular proliferation and induce differentiation (Tian *et al*, 1998). Antisense-EGFR transfection in rat C6 glioma cells was also found to inhibit cellular proliferation and induce apoptosis (Pu *et al*, 2000). However, the effects of antisense-EGFR on telomerase activity and telomere length are still unknown.

## MATERIALS AND METHODS

### Construction of antisense-EGFR vector

The construction of the antisense-EGFR construct and the cloning of the transfected cells have been described previously (Tian *et al*, 1998). The EGFR cDNA fragment (*Bam*HI-*Bam*HI) derived from *PE7* was inserted in reverse orientation at the *Bam*HI site of the *pBabe-puro* vector (Morgenstern and Land, 1990). This cDNA corresponds to the last 256 amino acid residues of the extracellular domain, the entire transmembrane domain and the first 61 amino acid residues of the cytoplasmic domain of EGFR.

### Cell culture and transfection

The human glioblastoma cell line U87MG (American Type Culture Collection, Rockville, MD, USA) was grown in Minimum Essential Medium-alpha (MEM-α) medium (Gibco, Grand Island, NY, USA) supplemented with 10% foetal bovine serum, 100 μg ml^−1^ streptomycin and 100 U ml^−1^ penicillin, in a humidified atmosphere of 5% CO_2_ at 37°C. Cells were transfected with the antisense-EGFR constructs using the Transfectam reagent (Promega Corp., Madison, WI, USA). Clones stably expressing undetectable or low levels of EGFR protein (AS-1, AS-3), were selected in 1 μg ml^−1^ puromycin (Sigma Chemical Co., St. Louis, MO, USA) as described previously (Tian *et al*, 1998). U87MG cells were also transfected with pBabe-puro vector to serve as a control.

### Telomeric repeat amplification protocol (TRAP) assay

TRAP-eze telomerase detection kit (Oncor) was used for telomerase extraction and TRAP assay according to the manufacturer's protocol with minor modifications as previously described (Chong *et al*, 1998). The TRAP assay was performed in a 12.5 μl reaction mixture containing cell extract (5, 1, 0.2, or 0.04 μg), 0.05 mM dNTP, ^32^P-end-labelled TS primer (5′AATCCGTCGAGCAGAGTT3′), primer mix (including a primer K1 and a template TSK1 for amplification of a 36-bp internal standard) and 0.5 unit of Taq DNA polymerase (Perkin-Elmer Cetus). After 20 min incubation at 30°C in the thermocycler block to allow telomerase-mediated extension of the TS primer, 30 cycles of PCR was performed at 94°C for 30 s, 58°C for 30 s, and 72°C for 30 s. Incorporation of the PCR internal standard allows identification of false-negative samples that may contain Taq polymerase inhibitors. The PCR products were examined by electrophoresis on a 12.5% non-denaturing polyacrylamide gel. Quantification of samples was performed using the InstantImager (Packard, Meriden, CT, USA) for electronic autoradiography. A heat-inactivated (85°C for 10 min) telomerase control, 1×CHAPS lysis buffer only control, and the TSR8 quantification control were included for each set of TRAP assays according to the manufacturer's instruction. The TRAP assay was repeated twice for each sample.

### Measurement of terminal restriction fragment (TRF) length by Southern blot hybridisation

Terminal restriction fragment (TRF) length, which is a measure of telomere length, was measured by Southern blot hybridisation. High-molecular-weight DNA was prepared from cultured cells using the standard procedures, including proteinase K treatment and phenol/chloroform extraction. Three micrograms of DNA from each cell line was digested overnight with the restriction endonuclease *Hae*III (Amersham Pharmacia Biotech, Buckinghamshire, UK). Electrophoresis through a 1% agarose gel and Southern transfer to a nylon membrane were performed as previously described (Counter *et al*, 1992). The synthetic oligonucleotide (TTAGGG)_4_ was end-labelled with [γ-^32^P]ATP and used as a hybridisation probe to detect telomeric repeats. Hybridisation was carried out at 50°C for 2 h in Rapid-hyb buffer (Amersham Pharmacia Biotech). This membrane was finally washed at 42°C in 2×SSC/0.1% SDS (1×SSC: 0.15 M NaCl and 0.02 M sodium Citrate), then autoradiographed. The densitometric peak of smear signal was defined in this study as the peak TRF length. Quantification of signals was carried out using Fluor-S MultiImager (Bio-Rad Laboratories).

### Tumorigenicity study of antisense-EGFR transfected cells

Balb/c nude mice, 6–8 weeks old, obtained from the Animal Resources Center, Western Australia, were housed in pathogen-free facilities. Ten mice were divided into four groups: two each for U87MG parental cells and empty vector-transfected cells and three each for AS-1 and AS-3 clones. Five million viable cells from each cell line were injected subcutaneously into the flank of each nude mouse of the relevant group. Tumour diameters in 3 dimensions were measured with linear calipers 1 week after inoculation, and tumour volumes were calculated as their product. This experiment was performed twice.

## RESULTS

### Human U87MG glioma cells successfully transfected with antisense-EGFR

The antisense-EGFR constructs were transfected into a glioblastoma cell line U87MG and stable transfected clones were isolated. The effect of antisense EGFR RNA on the expression of EGFR protein was evaluated by Western blot analysis using a rabbit anti-human EGFR polyclonal antibody. Parental cells and cells transfected with empty vector (denoted as U87MG/pBabe) expressed high level of EGFR protein. The protein level of EGFR was reduced about five-fold in antisense-EGFR clone AS-3, and was undetectable in antisense-EGFR clone AS-1. These successfully transfected antisense-EGFR clones were found to have impaired proliferation, reduced transforming potential as well as cellular differentiation (Tian *et al*, 1998). In this study, antisense-EGFR clones AS-1 (the most inhibited clone) and AS-3 (the intermediately inhibited clone) were further analysed in subsequent experiments. U87MG/pBabe cells were used as a control.

### Telomerase activity

**Figure 1:**
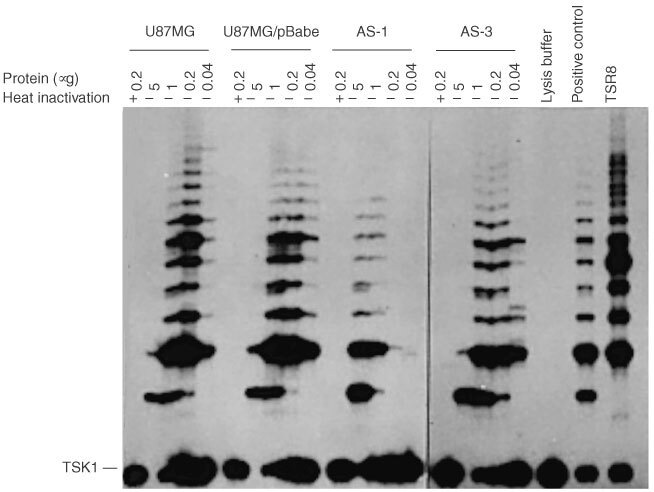
Telomerase activity in U87MG cells, empty vector-transfected cells (U87MG/pBabe), antisense-EGFR transfected cells AS-1 and AS-3. There was no significant difference in the telomerase activity between parental and empty vector-transfected cells. However, the telomerase activity reduced 54 folds and 2.5 folds in AS-1 (*P*<0.001) and AS-3 (*P*<0.05), respectively, when compared to that of the control cells. The negative control was lysis buffer and the positive control was provided by the TRAP-eze telomerase detection kit. TSR8 (control template) was used as a quantification control. TSK1 was the internal standard of PCR.

Telomerase activity, depicted by the incremental 6-nucleotide ladder, was detected in both empty vector-transfected and parental cells (Figure 1

). Pretreatment of cell extracts with heat abolished the laddering patterns, confirming the specific detection of telomerase activity. The cell extracts appeared to contain inhibitory activity against PCR reaction, as revealed by the absence of internal control TSK1 signal, when 5 μg of protein was used. The extension of the characteristic 6-base ladder and the appearance of the TSK1 band occurred after dilution of the cell extracts. In control cells, telomerase activity was detected from 1 to 0.04 μg (5–125-fold diluted) protein extract (Figure 1). In antisense-EGFR clone AS-1, the 6-base ladders were much weaker at 1 and 0.2 μg extract than the corresponding protein concentrations in control cells, and the activity was undetectable in 0.04 μg protein extract. A quantitative assessment of telomerase activity was performed by comparing the intensity of the 6-base ladders from 0.2 μg cell extracts of AS-1 and AS-3 to that of the control (TSR8). The experiment was performed three times. There was no significant difference in telomerase activity between parental cells and empty vector-transfected cells. However, telomerase activity decreased 54-fold and 2.5-fold in AS-1 (*P*<0.001) and AS-3 (*P*<0.05), respectively, when compared to that of the control cells. These results showed a strong correlation of telomerase activity with the expression levels of EGFR, indicating a role of EGFR in the regulation of telomerase activity.

### TRF length in antisense-EGFR transfected cells

**Figure 2:**
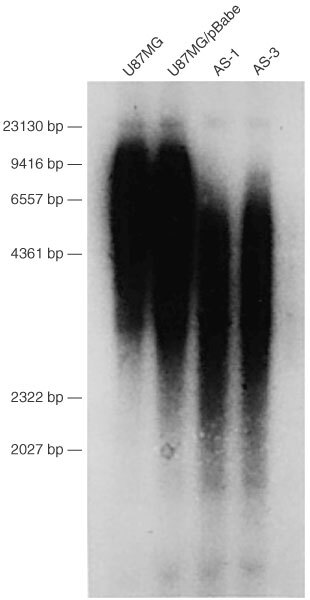
TRF length analysis by Southern blot of *Hae*III-digested DNA with the telomere-specific probe (TTAGGG)_4._ The densitometric peak in smear signal was taken as the peak TRF length. The peak TRF lengths were 7.6, 7.2, 3.8 and 4.1 kb in parental U87MG cell, empty vector-transfected cells (U87MG/pBabe), antisense-EGFR transfected clones AS-1 and AS-3, respectively. The peak TRF lengths of control cells and antisense-EGFR transfected cells correlated well with the telomerase activity.

Same passages of control cells and antisense-EGFR transfected cells were used to analyse TRF length. Figure 2

shows the hybridisation of the (TTAGGG)_4_ probe to *Hae*III-digested DNA from control cells and antisense-EGFR transfected cells. The densitometric peak in smear signal was taken as the peak TRF length. The peak TRF lengths were similar in parental U87MG cells (7.6 kb) and empty vector-transfected cells U87MG/pBabe (7.2 kb), whereas the peak TRF lengths were shortened in antisense-EGFR clone AS-1 (3.8 kb) and AS-3 (4.1 kb). AS-1, which expressed lowest telomerase activity, had the shortest peak TRF length. This experiment was performed twice and similar result was obtained. Thus, the peak TRF length correlated well with the telomerase activity of U87MG cells.

### Tumorigenicity

We tested whether the expression of antisense-EGFR RNA had any effect on the ability of glioblastoma cells to form tumours in athymic nude mice. Control cells and antisense-EGFR transfected cells were injected into nude mice subcutaneously, and tumour volumes were measured for the duration of the experiments. At 3 weeks of post-implantation (Table 1

**Table 1:**
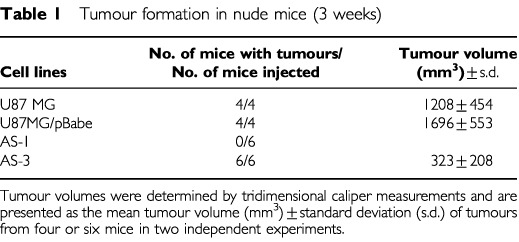
Tumour formation in nude mice (3 weeks)

), examination of nude mice revealed no significant difference in tumour volumes between U87MG and vector alone cells, and the control sections of the experiments were terminated. At this time point, however, AS-3 cells formed smaller tumours (*P*<0.001) in nude mice than control cells, and AS-1 had not yet produced visible tumours. Therefore, the monitoring of the nude mice injected with antisense-EGFR transfected cells was continued until tumour volumes reached ∼1000 mm^3^. AS-3 cells, expressing intermediate levels of EGFR, formed tumours of ∼1000 mm^3^ after 4 weeks post-implantation. Whereas, AS-1 cells, expressing the lowest levels of EGFR, required 16–18 weeks, to grow to a similar extent. There were strong correlations between tumorigenicity and EGFR expression levels, and between tumorigenicity and telomerase activity.

## DISCUSSION

In this study, glioblastoma U87MG cells that were previously shown to over-express EGFR, were transfected with the antisense-EGFR constructs. The stable transfectants expressed low or undetectable levels of EGFR protein (Tian *et al*, 1998). These transfected cells were found to be weakly- or non-responsive to epidermal growth factor induced cell growth (Tian *et al*, 1999). Although the mechanism of action is not clear, the antisense approach used in this study demonstrated high specificity toward inhibition of endogenous EGFR expression. These transfected cells were suppressed in cell growth *in vitro*, increased in G1 arrest and decreased in Ki-67 staining (Tian *et al*, 1998).

Activation of telomerase, compensating for the loss of telomeres, has been implicated in human cell immortalisation and carcinogenesis. Three human telomerase-associated molecules, human telomerase RNA (hTR), human telomerase catalytic subunit (hTERT) and telomerase-associated protein (hTEP1), have been identified. Its RNA subunit acts as a template for the synthesis of telomeric DNA, whereas hTERT catalyses this process to make up for the inability of conventional DNA polymerase to replicate completely the ends of linear DNA. hTEP1 is associated with telomerase activity and the telomerase reverse transcriptase, and it specifically interacts with the telomerase RNA (Dhaene *et al*, 2000). Wu *et al* (1999) has demonstrated that c-*myc*, a down-stream target of EGFR, can directly activate telomerase by inducing expression of the catalytic subunit of hTERT.

There were only two studies reporting an association of EGFR with telomerase activity. Kunimura *et al* (1998) dissociated primary human epithelial cells of uterine cervix into several distinctive cellular subsets by immunocytochemical cell fractionation. They found that telomerase activity was positive in the subset which expressed predominantly integrin beta 1 and EGFR, but was negative in the subset which strongly co-expressed p75NGFR, integrin beta 4 and bcl-2. Their work showed a phenomenon that EGFR expression and telomerase activity co-existed in the subset. In a mouse model, Inui *et al* (2002) demonstrated that after partial hepatectomy regenerating hepatocytes showed upregulation of telomerase activity. They further showed that preoperative treatment of EGF increased the *in vivo* telomerase activity. Such an increase in telomerase activity was also demonstrated in regenerating hepatocyte culture treated with EGF. Moreover, treatment with MEK inhibitors significantly repressed telomerase activity. Their findings suggest that EGF plays an important role in the activation of telomerase activity in liver regeneration. In this study, antisense-EGFR transfected cells expressed much lower telomerase activity than control cells did. AS-3 cells, which expressed intermediate level of EGFR, exhibited much higher telomerase activity than AS-1, which expressed the lowest level of EGFR. Thus, a direct correlation was observed between the levels of EGFR expression and telomerase activity. Our results show that EGFR is associated with regulation of telomerase activity in glioma cells, although the mechanism is currently unclear.

Telomerase activity has been shown to be specifically expressed in immortal cells, cancer cells and germline tissues, where it compensates for telomere shortening during DNA replication and thus stabilises telomere length (Dhaene *et al*, 2000). Our results showed that empty vector-transfected cells had the same peak TRF length as the parental U87MG cells, suggesting that the high telomerase activity in control cells can stabilise telomere length. Antisense-EGFR transfected cells, which expressed low telomerase activity, exhibited shorter peak TRF length than control cells, this result is in keeping with the role of telomerase for maintenance of telomere. Murakami *et al* (1999) used reverse transcriptase inhibitors, dideoxyinosine (ddI) and AZT-5′ triphosphate (AZT-TP), to inhibit telomerase activity of gynaecological cancer cells. They found that ddI and AZT-TP treatment of tumour cells reduced telomerase activity, shortened the length of the telomere and increased p53 expression. Hahn *et al* (1999) exhibited that expression of a mutant catalytic subunit of human telomerase resulted in complete inhibition of telomerase activity, reduction in telomere length, death of tumour cells and elimination of tumorigenicity *in vivo*. Our study demonstrated that antisense-EGFR approach could also suppress telomerase activity in glioma cells. Antisense-EGFR transfection of U87MG cells resulted in inhibition of telomerase activity and tumorigenicity, reduction in telomere length, increase in p53 expression (Tian *et al*, 1999) and apoptosis induced by cisplatin (Tian *et al*, unpublished data). There were strong correlations between tumorigenicity and EGFR expression levels, and between tumorigenicity and telomerase activity. Thus, the finding that antisense-EGFR transfection induces a decrease in telomerase activity and telomere length suggests a novel mechanism of antisense-EGFR treatment in inhibiting cellular proliferation.

In an attempt to elucidate the mechanism of EGFR in regulating telomerase activity, we evaluated the transcriptional levels of c-*myc*, hTERT and hTEP1. Semiquantitative RT-PCR assay showed no significant difference in the transcriptional levels of c-*myc*, hTERT and hTEP1 between antisense-EGFR cells and control cells (data not shown). Many research groups have demonstrated that telomerase activity correlates well with hTERT expression, but not with hTEP1 expression (Dhaene *et al*, 2000). Recently, a few reports showed that hTERT expression was not related with telomerase activity (Kameshima *et al*, 2001; Liu *et al*, 2001). Actually, the mechanisms regulating the production of active telomerase enzymes are still predominantly unknown, although roles for transcriptional control of hTERT, alternative-splicing of hTERT transcripts (Ulaner *et al*, 2000) and post-transcriptional phosphorylation of hTERT have been advocated (Kang *et al*, 1999). Kiaris and Schally (1999) reported that telomerase activity decreased in U87MG cells after treatment with an antagonist of growth hormone-releasing hormone MZ-5-156. The authors found that the expression of hTERT was significantly decreased by MZ-5-156, whereas the levels of mRNA for hTR and hTEP1 were unaffected. Moreover, the repression of the telomerase activity was not accompanied by a significant decrease of mRNA level for the c-*myc* protooncogene that regulates telomerase (Kiaris and Schally, 1999). Our results are in agreement with Kiaris's, indicating that c-*myc* may not be involved in the regulation of telomerase activity in U87MG cells. EGFR may regulate the telomerase activity through other downstream molecules, but not through c-*myc*. The signal transduction pathway of EGFR is complex. EGFR can also activate protein kinase C (PKC), which is a family of ubiquitous serine/threonine protein kinases involved in growth control and tumorigenesis. Li *et al* (1998) reported that phosphorylation of hTERT and hTEP1 by protein kinase C alpha was an essential step in the activation of telomerase complex. In U87MG cells, it is possible that EGFR up-regulates telomerase activity through phosphorylation of hTERT and hTEP1 by protein kinase C alpha, but not through transcriptional increase of hTERT and hTEP1. The mechanism of EGFR regulating telomerase activity remains unclear.

In conclusion, this study provides evidence that EGFR plays an important role in the regulation of telomerase activity of glioma cells. Our findings provide new insights into both the biological functions of EGFR and the regulation of telomerase activity. The inhibition of telomerase activity triggered by antisense-EGFR treatment may reflect yet another mechanism of antisense-EGFR approach in tumour suppression.
